# ‘A tool for every job’: use of video in urgent primary care

**DOI:** 10.3399/bjgp24X739473

**Published:** 2024-09-27

**Authors:** Ulrik Bak Kirk, Rebecca Payne, Jaime-ann Tweedie, Linda Huibers

**Affiliations:** Department of Public Health, Aarhus University, Aarhus, Denmark; Research Group Leader, Digital Health Research, Research Unit for General Practice, Aarhus, Denmark.; Nuffield Department of Primary Care Health Sciences, University of Oxford, Oxford, UK.; National Medical Lead, Integrated Urgent Care-contact (111) for Practice Plus Group, Reading, UK; Emergency Operations Centre GP, South Western Ambulance Service NHS Foundation Trust (SWAST), Bristol, UK.; Research Group Leader for Acute Primary Care Research, Research Unit for General Practice, Aarhus, Denmark.

Telemedicine, including video consulting, has been available for decades and its widespread adoption accelerated dramatically during the COVID-19 pandemic.^1^ This surge was driven by the need to ensure continued access to health care while minimising the risk of viral transmission. There is a growing body of evidence regarding video consultations, but much of the research focuses on secondary care settings and specific sub-populations, such as those with diabetes.^2^ Despite studies from Denmark,^3^ Norway,^4^ Sweden,^5^ and the UK,^6^ a lack of evidence remains regarding the use of video in primary care, particularly outside office hours for patients seeking urgent care.^7^^,^^8^

As life returns to a new normal after COVID-19, the momentum behind video consultations in general practice appears to have waned.^3^^,^^6^ This decline can be attributed to concerns regarding the lack of face-to-face interaction, technical challenges, and challenges conducting physical examinations remotely.^7^ Consequently, video consultations are now rarely used in routine general practice.^6^ However, there is growing appreciation that video consultations can complement telephone consultations in the out-of-hours primary care setting. Many organisational models exist, using a range of healthcare professionals.^9^ Here video can serve both as an alternative to face-to-face consultations and provide a plethora of additional information, particularly in the context of third-party consultations.^7^

## Complementing out-of-hours primary care

In the out-of-hours primary care services, the patient and clinician are usually unknown to each other. Such care is inherently more transactional in nature than relational, as it often focuses on an urgent single health problem. Thus, while video consultations may be perceived as a step down from a face-to-face consultation at the in-hours setting, they can form a helpful addition to telephone assessment in the context of out-of-hours care, for clinicians working at these services. The additional information gathered via video can reduce consultation length by reducing the number of questions to be asked — ‘a picture can say a thousand words’. This information can support the clinician’s decision-making process and facilitate direct referral to an appropriate service or a safe completion of the consultation. Furthermore, video consultation can be complemented by the use of the patient’s own technology, such as pulse oximeters, thermometer, and photographs showing, for example, the evolution of a lesion. Though the ubiquity of mobile devices and apps that integrate video consultation capabilities offer convenience and immediacy, particularly valuable in urgent situations where time is of the essence, patients increasingly expect 24-hour access to services. This 24/7 accessibility also raises concerns about the potential for over-reliance on technology and over-use of video for some patients in situations where a face-to-face consultation might be more appropriate.

Where clinically appropriate, replacement of a face-to-face consultation by video consultation is particularly appreciated by patients in the out-of-hours setting who otherwise might have to travel long distances. Video consultations seem to demonstrate benefits in cases involving injuries, skin conditions, respiratory infections, or fever, especially in paediatric patients.^10^ However, image quality is unlikely to be of the same standard as that obtained on still photos in services such as teledermatology, particularly when patients are joining calls from mobile phones.^11^ In addition, distortions of colour can cause conditions such as cyanosis or jaundice to be missed.^12^

## Addressing safety considerations

Safety incidents are rare in all forms of remote consulting, but sadly do occur.^12^ Tragic incidents have been highlighted in the media, contributing to a narrative suggesting that video consultations may not be suitable to replace a face-to-face consultation in general practice.^13^ A more balanced media approach, highlighting that the benefits likely outweigh risk, is unfortunately lacking. The authors recognise that the rapid integration of video into clinical practice has not been complemented with standardised guidelines. Healthcare professionals are therefore left to make their own decisions regarding appropriate use of video, based on their own experience and the nature of the patient’s concern.^14^

Video is considered inappropriate for the examination of intimate areas, and less useful for the assessment of abdominal pain, ears, and cardiac issues, although being able to swiftly ‘eyeball’ such patients can bring benefit on deciding on next steps and timescale for further assessment.^12^ Training is needed for staff providing care via video, and appropriate infrastructure needs to be in place, including headsets and dual screens. Patients will require broadband or mobile data signals to connect, which can often be challenging in rural areas, where the technology can otherwise bring most value.

Given the utility of the additional information video brings to the out-of-hours primary care setting, service providers and professional bodies should consider providing clear guidance for the safe and appropriate use of video consultation. Where clinicians choose to deviate from such guidance, their rationale should be clearly documented.

The authors suggest that in order to fully realise the potential of video, particularly as a triage tool in out-of-hours primary care, such guidelines should incorporate the following:

- a range of options for video use (1-way versus 2-way, complex versus ‘click-a-link’ and so on);
- the roles of video clearly defined, for example, a tool in urgent telephone triage versus remote consultation; and
- evidence-based guidelines for when video should be used to add value to patient care, and when video should be used with caution.

By establishing clear guidelines and recommending evidence-based practice, the positive aspects of video use in primary care can be harnessed, while its limitations are acknowledged and addressed. This approach will ensure that clinicians include video in their consultation toolkit and have the skills to appropriately choose video to provide safe and effective individual patient care.
